# A multidimensional ensemble pipeline for early detection of IUGR condition through CTG

**DOI:** 10.3389/fdgth.2026.1837088

**Published:** 2026-06-15

**Authors:** Edoardo Spairani, Giulio Steyde, Francesco Podda, Maria G. Signorini, Giovanni Magenes

**Affiliations:** 1 Department of Electrical, Computer and Biomedical Engineering, University of Pavia, Pavia, Italy; 2 Centre for Health Technologies (CHT), University of Pavia, Pavia, Italy; 3 Department of Electronics, Information and Bioengineering (DEIB), Politecnico Milano, Milano, Italy

**Keywords:** antepartum cardiotocography, artificial intelligence, deep learning, ensemble learning, IUGR

## Abstract

**Introduction:** Intrauterine growth restriction (IUGR) is a major cause of perinatal morbidity and mortality, often associated with placental insufficiency and progressive alterations in fetal autonomic regulation. Cardiotocography (CTG) represents one of the most widely used tools for fetal monitoring, yet its interpretation remains challenging due to high inter-observer variability and the subtle nature of early pathological patterns. Artificial intelligence approaches have recently shown promising potential for automated CTG analysis, but their development is often limited by the scarcity of large, annotated datasets.

**Methods:** In this study we propose a multidimensional ensemble pipeline for the detection of IUGR from antepartum CTG recordings. The framework integrates two complementary predictive branches: a residual deep learning model (ResNet) operating directly on multivariate temporal sequences, and a hybrid CNN–MLP architecture combining image-based encodings of fetal heart rate signals with physiologically interpretable quantitative descriptors. The outputs of the two models are fused through a logistic regression meta-classifier using a stacking strategy. The pipeline was trained and evaluated using the NAPAMI database, a large clinically curated dataset comprising more than 70,000 CTG recordings collected over a period of 17 years.

**Results:** Both base models (ResNet and CNN + MLP) achieved comparable performance levels. The proposed ensemble approach significantly improved the overall performance, reaching a balanced accuracy of 0.799 and an AUC of 0.868 (95% CI: 0.849–0.885). Statistical comparison using McNemar's test confirmed that the ensemble classifier significantly outperformed the individual models (*p* < 10^−11^).

**Discussion:** The results demonstrate that combining complementary representations of fetal heart rate dynamics through an ensemble framework can improve the detection of IUGR from antepartum CTG recordings. The use of a large-scale clinical dataset together with physiologically informed and deep learning-based representations provides a promising direction for the development of AI-assisted decision support tools in prenatal medicine.

## 1 Introduction

### 1.1 Background

The antepartum period represents a critical phase for fetal health, during which appropriate monitoring before delivery plays a key role in preventing complications and improving perinatal outcomes (1). Reliable assessment of fetal well-being is therefore essential across a wide range of clinical scenarios, ranging from acute conditions such as maternal bleeding or abdominal pain to chronic disorders including preeclampsia, gestational diabetes, and impaired fetal growth (2). The main objective of antepartum monitoring is the early identification of any fetal compromise in order to reduce the risk of adverse prenatal and postnatal outcomes (3).

Cardiotocography (CTG) remains one of the most widely used non-invasive techniques for fetal surveillance, providing simultaneous recordings of fetal heart rate (FHR) and uterine contractions.

In the early stages of its introduction, CTG interpretation based on visual inspection of FHR patterns (4), significantly contributed reducing fetal morbidity and mortality during labor, even if considerably affected by inter-observer variability among clinicians.

Although it is known that FHR variations are closely related to fetal autonomic regulation and therefore represent an important indicator of fetal well-being throughout pregnancy (1–5), the usefulness and the reliability of CTG monitoring and analysis during the antepartum period are still controversial (2).

Early research efforts aimed at supporting clinical interpretation through quantitative analysis of FHR signals, introducing statistical descriptors such as accelerations and decelerations, computed on a baseline estimation, and variability indices (6–9). These descriptors include Short-Term Variability (STV), Long-Term Irregularity (LTI), Delta, and Interval Index, which are the widely used features in computerized CTG analysis (7).

However, these classical descriptors capture only morphological and time-related features of fetal heart rate dynamics. Studies on heart rate variability have shown that cardiac rhythm results from complex interactions between the sympathetic and parasympathetic nervous systems, influenced by both central and peripheral regulatory mechanisms (10, 11). This evidence led to the introduction of more advanced signal analysis techniques, including spectral analysis and nonlinear complexity measures, which enable a more comprehensive characterization of fetal heart rate dynamics (12). Entropy-based and complexity metrics have demonstrated their ability to capture subtle physiological alterations associated with pathological conditions, including fetal distress and intrauterine growth restriction (IUGR) (13, 14).

As a matter of fact, IUGR represents one of the most challenging complications of pregnancy since it affects approximately 5%–10% of pregnancies and is associated with increased perinatal morbidity and mortality. The condition is typically linked to placental insufficiency, which progressively alters fetal oxygenation and autonomic regulation, potentially producing subtle changes in fetal heart rate dynamics.

Early identification of IUGR during pregnancy is of particular importance because it directly influences clinical decision-making about the delivery time. In IUGR cases, timely intervention may prevent further deterioration of fetal conditions and reduce the risk of severe complications (15). The IUGR condition is usually diagnosed on the basis of fetal Doppler Pulsatility Index (PI) abnormalities, in combination with the estimated fetal weight (EFW) (16). However, a quite recent study, which included neonatal follow-up, demonstrated that a combination of Doppler measurements and advanced CTG parameters improves the diagnosis of IUGR (17). For this reason, the role of CTG analysis become relevant during the antepartum phase of pregnancy, when the detection of early physiological alterations can be supported by preventive clinical strategies (18). Several studies considered FHR variability features to identify growth-restricted fetuses. Nonlinear indices have shown promising results in distinguishing severe IUGR cases from healthy fetuses (14, 19, 20). More recently, ML approaches have also been explored for the identification of IUGRs using FHR-derived features: Signorini et al. (21) adopted linear and nonlinear FHR indices extracted from a selected population of both healthy and IUGR fetuses, which enable the evaluation of ML models for discriminating the two groups (22). Building on this dataset, subsequent studies have investigated the application of machine learning algorithms for predicting late IUGRs using CTG features (23). However, in real clinical practice, the identification of IUGRs on the basis of prenatal CTG recordings remains a critical challenge.

### 1.2 State of the art

In recent years, artificial intelligence techniques, including both ML and deep learning (DL), have increasingly been explored for automated CTG interpretation and classification. DL architectures, particularly convolutional neural networks (CNN), have shown promising performance in fetal state classification and CTG signal analysis tasks (24–28), as they are capable of automatically learning discriminative representations from raw physiological signals without relying on overwhelming sets of indices (29).

Most research utilizing DL on CTG signals has primarily focused on detecting fetal distress (in particular acidemia) and/or fetal sufferance conditions by means of DL methods applied to FHR signals (24–28). Unfortunately, the majority of them have made use of CTG tracings recorded during labor, which depict the fetal condition very close to delivery and thus are not viable for an early detection of IUGRs.

Several recent works that have adopted DL methods have addressed the binary classification of CTG tracings based on whether they belong to healthy or pathological fetal classes. For example, Cao et al. (26) proposed a DL framework combining CTG signals and clinical indices to discriminate between normal and abnormal tracings. In their dataset, suspicious and pathological cases were grouped into a single abnormal class, resulting in a binary classification problem. The dataset (26) included 11,998 normal cases, 4,326 suspicious cases and 31 pathological cases. The classifications were assigned based on obstetrician assessments of the CTG traces, in accordance with established clinical guidelines, and/or based on confirmed neonatal outcomes. Under these conditions, the model accuracy was 90.77% and the AUC = 0.9201.

Similarly, Fei et al. (28) proposed a multimodal DL architecture based on bidirectional gated recurrent units for the classification of antenatal CTG recordings into healthy and pathological classes, reporting an accuracy of 86.45% and an AUC of 0.9327.

Other studies have explored different modelling paradigms for CTG analysis (30, 31). For instance, Kong et al. (32) investigated the use of ensemble machine learning techniques to identify fetal hypoxia or acidosis during labour. Their experiments were performed on the CTU-UHB intrapartum dataset (33), which contains 502 recordings.

Ahmed et al. (34) proposed an ensemble framework which integrates multiple machine learning and deep learning models in a stacking classifier for CTG categorization. The reported performance metrics were extremely high (accuracy of 98.9% and AUC of 99.8%); however, the experiments were done on the UCI CTG dataset, which contains 2,126 samples represented by 23 precomputed statistical features derived from fetal heart rate signals rather than from raw CTG recordings.

More recently, transformer-based architectures have also been investigated. Khan et al. (35) introduced PatchCTG, a transformer-based model designed to classify CTG recordings into adverse pregnancy outcome (APO) vs. normal pregnancy outcome (NPO). The model achieved an AUC of 0.77 (sensitivity 57%, specificity 88%). In the study, the target outcome corresponded to a composite endpoint including several heterogeneous clinical conditions. The CTG recordings were taken during the seven days prior to delivery.

Another interesting study related to fetal hypoxia identification was recently proposed by Mohan et al. (36), who developed an attention-based 1DCNN-BiLSTM model for intrapartum fetal monitoring. Their approach combines FHR signal preprocessing, DWT-based denoising, SMOTE-based data balancing, sliding-window segmentation and an attention-based hybrid deep learning architecture to classify fetal hypoxia. Although the study reported high classification performance, its objective was the detection of intrapartum hypoxia using CTU-UHB recordings and therefore differs substantially from the antepartum IUGR prediction task.

In the scientific literature there are relatively few studies focusing on IUGR prediction leveraging CTG signals and DL. A systematic review and meta-analysis by Rescinito et al. (37) examined 20 studies exploring the use of AI models for the prediction of IUGR based on heterogeneous data sources, including CTG signals, biochemical biomarkers, Doppler indices and genomic information. The pooled analysis reported an overall sensitivity = 0.84 and specificity = 0.87, while the accuracy reported across individual studies ranged approximately from 79% to 97%, depending on the adopted algorithm and input modality.

The development of robust AI models for CTG, however, is strongly influenced by the availability and quality of annotated datasets. A recent review of AI methods applied to CTG analysis (38), which considered 88 studies published between 2013 and 2024, highlighted that 42% of the studies relied on private datasets, while 31% used the CTU-UHB dataset (33), 17% used the UCI dataset (39), and only 2% used the SysPorto dataset (40).

Despite their undoubted importance, these datasets present several limitations, including relatively small sample sizes, lack of raw signals in some cases (38), and strong class imbalance. Therefore, the generalizability of many proposed methods remains difficult to assess. Moreover, a significant portion of publicly available CTG datasets consists of intrapartum recordings, which are acquired during labor and reflect fetal conditions in close temporal proximity to delivery. While these data are highly informative for the detection of acute fetal distress, they are not suitable for the present study, which focuses on antepartum prediction of IUGR. In fact, intrapartum recordings capture short-term physiological responses immediately preceding birth, whereas antepartum assessment aims to identify growth restriction earlier in pregnancy, when clinical decisions regarding monitoring, timing of delivery, and intervention strategies must still be made. Consequently, models developed and validated on intrapartum data cannot be directly transferred to the antepartum setting, due to differences in physiological dynamics, clinical objectives, and label definitions.

Furthermore, models evaluated on small and highly curated datasets often report very high-performance metrics; however, when applied to larger and more heterogeneous clinical datasets, their performance frequently decreases, reflecting the increased variability of real-world physiological recordings. This phenomenon has also been reported in cross-database evaluation studies, where models trained on a given CTG dataset exhibit a significant drop in performance when tested on independent datasets characterized by different signal distributions and annotation criteria (41).

### 1.3 Study rationale

Addressing the aforementioned limitations, this paper proposes an AI-based ensemble framework aimed at identifying IUGR fetuses antepartum from CTG recordings, grounded on the creation of a real large-scale clinical dataset (NAPAMI), which includes more than 70,000 CTG labeled recordings collected over more than 10 years.

Rather than introducing a single novel model, in this work we aim to integrate several methodological approaches, previously developed by our research group, within a unified analytical framework, including behavioral state identification, multiscale feature extraction, image-based signal encodings, and deep residual neural networks for CTG analysis. The key contribution of this work is not in the introduction of a standalone novel model, but in the coherent integration of multiple complementary methodologies into a unified framework designed to address the challenges of real-world clinical settings.

The proposed pipeline combines two complementary predictive branches: a ResNet model applied to raw CTG temporal signals, and a hybrid CNN–MLP architecture that jointly processes image-based FHR encodings and physiologically interpretable quantitative features. The outputs of the two models are then combined through an ensemble learning strategy based on logistic regression to produce the final probability of an IUGR condition.

## 2 Materials and methods

### 2.1 Introduction to the NAPAMI dataset

The dataset used for the present experimental analysis was derived from the NAPAMI (NApoli PAvia MIlano) repository, a large-scale and clinically curated collection of antepartum CTG recordings developed by our research group. A first preliminary version of NAPAMI was presented in Spairani et al. (42).

The actual dataset comprises 71,052 CTG recordings from 13,121 pregnancies, including synchronized FHRs, uterine contraction signals (TOCO), and fetal movement profile (FMP) signals, together with structured clinical metadata and outcome-related annotations.

NAPAMI was conceived to provide a heterogeneous and clinically representative foundation for both observational studies in prenatal medicine and the development of AI-based predictive systems. Tracings were collected during routine non-stress tests at the Federico II University Hospital in Naples, Italy, in a period spanning from 2005 to 2022. All patients signed the informed consent to the collection of CTG recordings for research purposes and all data were anonymized before any processing. Each CTG session, recorded in a controlled clinical environment using standard monitoring devices, lasted between 20 and 60 min. Importantly, each recording is associated with clinical metadata that provide valuable context for interpretation. For each exam, information such as the mother's age, gestational age (GA) at the time of monitoring, and a free-text clinical note is available. This note field, filled by the clinician at the time of the exam, contains unstructured annotations that may include a wide variety of relevant information, such as the use of medications by the mother, the presence of specific maternal or fetal conditions, or, when the pregnancy outcome is known, the birth weight, Apgar score and cord blood gas analysis of the newborn. Although written in natural language, and thus requiring additional processing, this free-text field represents a rich source of additional features that can enhance both clinical interpretation and data-driven analysis.

Each recording is also labeled with a class code reflecting the clinical condition of the pregnancy. This labeling system, established by clinicians according to ACOG guidelines, identifies 12 possible categories, including physiological pregnancies, twin pregnancies, fetal pathologies such as IUGR, maternal diseases with potential hypoxic impact, and various combinations of these risk factors. As discussed in Section 2.6, the experiments reported in this study were conducted on a subset of the NAPAMI dataset, focusing on pregnancies labelled as IUGR.

However, it is important to note that the IUGR label is not homogeneous in terms of diagnostic certainty. A substantial fraction of recordings corresponds to pregnancies classified as presumed IUGR, based on antenatal clinical suspicion, for which postnatal confirmation is not always available (because the pregnant women did not deliver the baby in the same hospital). Consequently, this subgroup may include fetuses that are constitutionally small for gestational age but not affected by pathological growth restriction, introducing a degree of label uncertainty and potential noise in the positive class. This aspect may reduce the effective separability between classes and must be explicitly accounted for in both model development and interpretation of results (see Section 2.6).

### 2.2 The proposed pipeline

**Figure 1:**
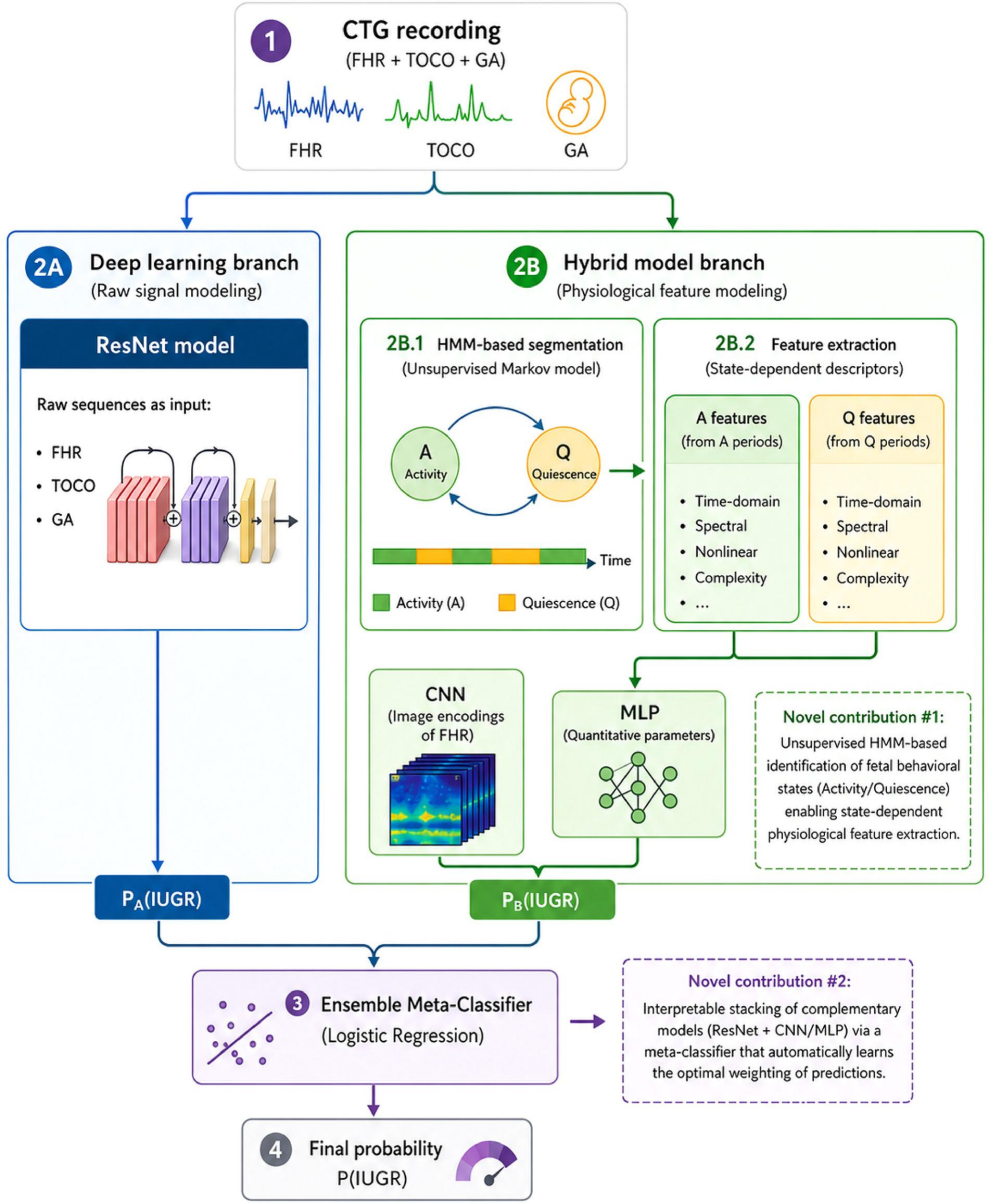
Scheme of the proposed pipeline.

The conceptual architecture of the proposed framework is schematically illustrated in Figure 1. Starting from a standard antepartum CTG recording, comprising fetal heart rate (FHR), uterine activity (TOCO) and GA, the information is processed through two parallel and complementary modelling streams. The first branch is a residual DL model (ResNet) operating directly on raw temporal sequences, thereby capturing complex temporal dependencies and nonlinear dynamics in a fully data-driven manner. The second branch is a hybrid architecture that combines a convolutional neural network (CNN), applied to image-based encodings of FHR signals, with a multilayer perceptron (MLP) fed by quantitative, physiologically interpretable parameters derived from the recordings.

Each branch independently produces an estimated probability of intrauterine growth restriction (IUGR). The two probabilities are then integrated by a logistic regression meta-classifier, which learns how to optimally weight the contribution of each model. The result is a single final probability that reflects the complementary information extracted by the two predictive branches.

Details regarding the two predictive branches and the ensemble meta-classifier will be illustrated in Sections 2.3–2.5.

Based on the characteristics of the dataset described above, the prediction of IUGR from antepartum CTG recordings is formulated as a binary classification problem under partial and asymmetric label uncertainty. Each recording is associated with a label *y* ∈ {0,1}, indicating healthy (0) or IUGR (1) condition. Importantly, the positive class includes both presumed and confirmed IUGR cases, which differ in terms of diagnostic reliability, resulting in a form of label noise primarily affecting the positive class. This aspect directly motivates the learning strategy adopted in this study, as detailed in Section 2.6.

### 2.3 Resnet model

**Figure 2:**
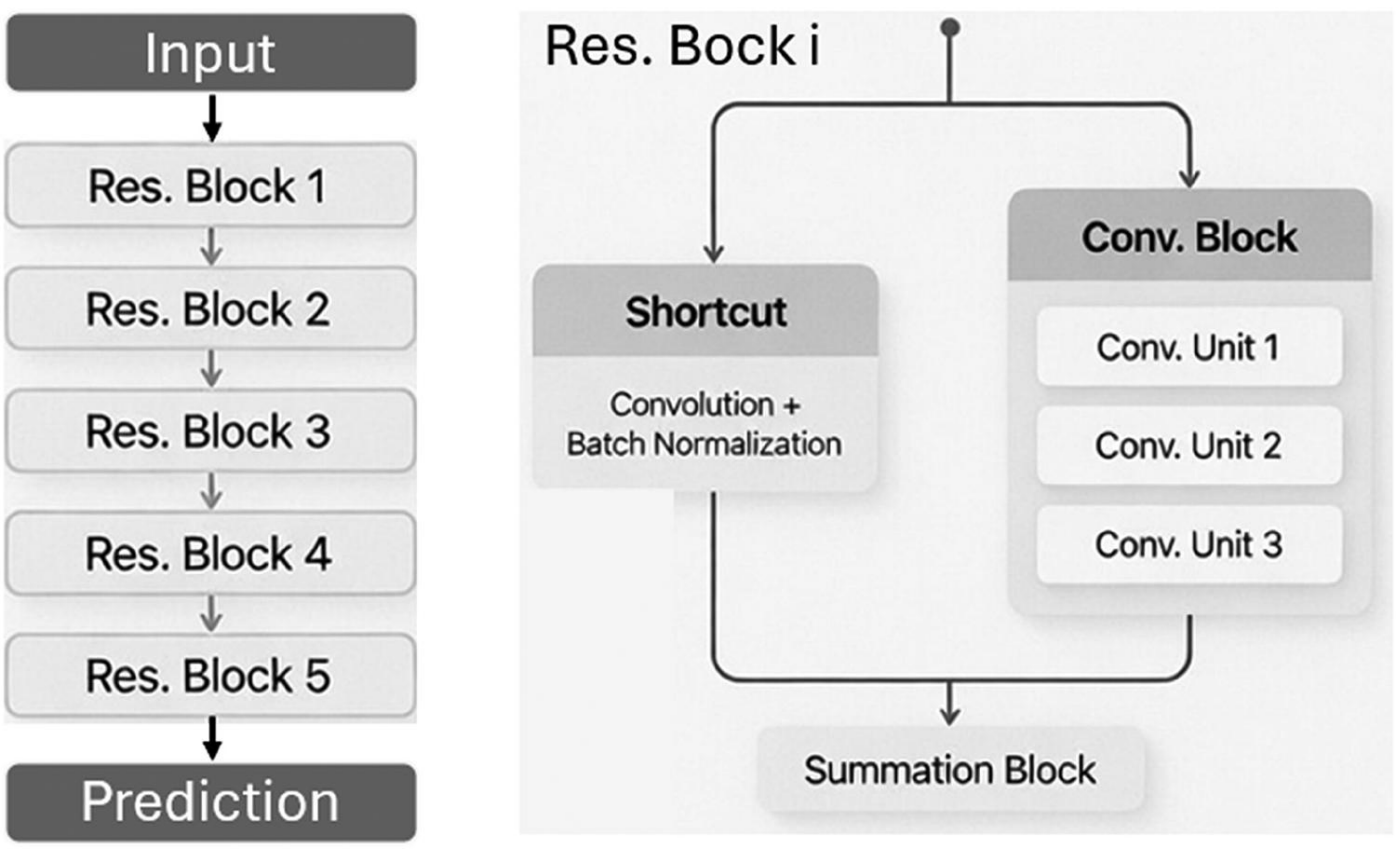
Scheme of the proposed ResNet model.

The classification ResNet model employed in this work was previously described in a paper from our research team (43). The network operates on fixed-length multichannel temporal segments of 20 min (i.e., 2,400 points) extracted from antepartum cardiotocographic recordings, incorporating FHR, TOCO, and GA information (this scalar is repeated 2,400 times to match the temporal dimensionality of the other channels). After standardized preprocessing and windowing, each sample is represented as a multivariate temporal tensor and processed through a cascade of five one-dimensional residual blocks composed of convolutional layers, batch normalization, nonlinear activations, and identity shortcut connections (Figure 2). This structure enables hierarchical feature extraction while preserving low-level signal information and ensuring stable optimization dynamics.

As will be detailed in Section 2.6, model training follows a two-stage strategy. An initial training phase leverages the larger subset of recordings labelled as presumed IUGR cases, allowing the network to learn robust and generalizable signal representations from a heterogeneous population. Subsequently, the model is refined through a fine-tuning phase performed exclusively on confirmed cases, i.e., recordings associated with neonatal outcome information. This second stage adjusts the higher-level decision layers considering labels characterized by greater diagnostic certainty.

This sequential training design allows extensive data utilization during representation learning, while progressively emphasizing high-confidence annotations in the final classification stage.

The final layer of the network outputs the estimated probability that a given recording belongs to the IUGR class. This probabilistic score is subsequently provided to the ensemble module, where it contributes to the refinement and calibration of the overall prediction.

Further architectural and implementation details of the model can be found in Spairani et al. (43).

### 2.4 Hybrid model

To better capture temporal dynamics and complex signal structures, while explicitly exploiting physiologically meaningful quantitative descriptors, we adopted a hybrid deep learning architecture inspired by the mixed-type dual-branch framework previously introduced by our research group in Spairani et al. (44). The overall structure remains consistent with that formulation: a Convolutional Neural Network (CNN) processes image-based encodings of the FHR signal, while a Multi-Layer Perceptron (MLP) processes quantitative clinical parameters.

In the present work, the CNN branch has been preserved without architectural modifications with respect to Ayres-de Campos et al. (40). It receives as input a set of grayscale images derived from the same 20-minute FHR segment using complementary transformations, including spectrograms, Continuous Wavelet Transform (CWT) scalograms, persistence spectra, recurrence plots, Markov Transition Fields, and Gramian Angular Fields (GADF) (44). These encodings emphasize different properties of the signal (time–frequency components, recurrence dynamics, correlation geometry, etc.) thus allowing the network to extract morphological patterns not readily accessible through traditional one-dimensional analysis.

The main extension introduced in the current study concerns the MLP branch.

In addition to the global quantitative descriptors originally employed, the MLP now receives state-dependent features computed separately within periods of fetal Activity (A) and Quiescence (Q).

Global parameters (18 features) include several complementary families of descriptors capturing different aspects of FHR dynamics. Time-domain statistics describe the overall distribution of the signal and include mean, standard deviation, median, and interquartile range. Acceleration and deceleration–related indices, derived from Phase Rectified Signal Averaging (PRSA), include acceleration capacity, deceleration capacity, and deceleration reserve. Spectral descriptors derived from PRSA quantify normalized power in low, medium, and high-frequency bands, reflecting autonomic modulation components of the FHR signal. Finally, nonlinear and complexity measures include Approximate Entropy, Sample Entropy, slope multiscale entropy, sample asymmetry, and Lempel–Ziv complexity (binary and ternary encodings). Together, these descriptors capture temporal variability, autonomic modulation in the frequency domain, and nonlinear dynamical properties of the signal, providing a comprehensive quantitative characterization of FHR regulation, as discussed in detail in Ayres-de Campos et al. (40).

In addition to these global descriptors, a second set of 22 parameters is computed separately within Activity and Quiescence phases (A and Q). These parameters are extracted from short temporal segments and capture state-dependent characteristics of FHR variability. They include time-domain descriptors (mean FHR and total signal power PWT), spectral power components (VLF, LF, MF, HF and their normalized percentages), nonlinear entropy measures computed at different embedding dimensions and tolerance levels (Approximate Entropy and Sample Entropy), complexity metrics such as binary and ternary Lempel–Ziv complexity, long-term variability indicators such as Long-Term Irregularity (LTI), and additional signal quality and phase-duration indicators.

These parameters are computed on sliding windows of 3 min (P3) and subsequently aggregated by averaging the values obtained within each behavioral phase (A and Q) across the entire recording.

This stratified aggregation allows the model to capture state-specific autonomic regulation patterns that may be diluted when descriptors are computed over the entire tracing without accounting for fetal behavioral state.

The identification of fetal Activity and Quiescence phases is performed through the unsupervised multivariate Hidden Markov Model (HMM) previously developed by our group and described in detail in Spairani et al. (45). The HMM models latent behavioral state transitions using clinically meaningful FHR-derived features and provides probabilistic segmentation of the tracing without requiring manual annotation. This segmentation step enables the systematic extraction of state-dependent parameters, thereby integrating fetal behavioral dynamics into the classification framework.

In this work, the HMM is intentionally used as a fixed upstream module rather than being embedded within the learning pipeline. The aim of this design is not to learn latent representations end-to-end, but to explicitly structure the input space in terms of physiologically interpretable behavioral states. This allows the model to learn whether differences in quantitative descriptors computed within Activity and Quiescence phases contribute to discriminating between healthy and IUGR conditions, instead of relying on abstract latent states that may be harder to interpret.

Furthermore, keeping the HMM decoupled from the classifier preserves the stability of the state estimation and avoids coupling behavioral state inference with the optimization dynamics of the classification task, which is performed under partial and asymmetric label uncertainty. This is particularly relevant in the presence of presumed IUGR cases, where jointly learning latent states and class labels could lead to less physiologically meaningful representations. Finally, the adopted modular design enhances reproducibility and robustness, allowing the same state segmentation procedure to be consistently applied across the entire dataset and enabling the explicit handling of missing behavioral phases. In fact, since not all recordings necessarily exhibit both behavioral phases within the analyzed window, a dedicated masking strategy was implemented. A binary flag vector of 44 elements (22 for A + 22 for Q), corresponding to the state-dependent feature set, is appended to the MLP input. For each feature, a value of 1 indicates valid computation, whereas 0 denotes absence due to missing behavioral phase. This vector allows the MLP to explicitly learn which portions of the input should be effectively masked, preventing spurious influence of undefined features and improving robustness across heterogeneous recordings. The final MLP input therefore consists of the concatenation of the 18 global descriptors, the 22 averaged Activity-derived parameters, the 22 averaged Quiescence-derived parameters, and the associated 44-element validity mask, for a total number of 106 features, forming an extended structured representation of fetal autonomic dynamics. The overall hybrid network thus preserves the original CNN-MLP architecture introduced in Spairani et al. (44), while extending the quantitative branch to incorporate state-aware physiological descriptors derived through the HMM framework of Spairani et al. (45).

**Figure 3:**
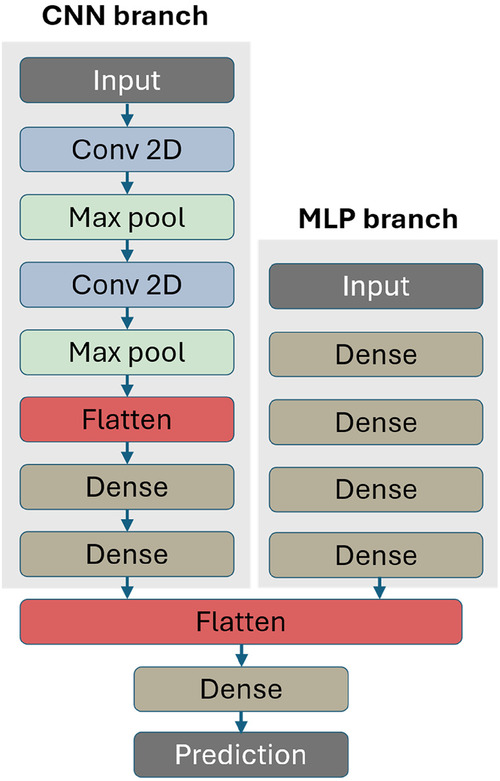
CNN + MLP hybrid model.

The schematic depiction of the proposed CNN + MLP architecture is illustrated in Figure 3.

### 2.5 Ensemble meta-classifier

To enhance the robustness and generalizability of the final prediction, a stacking-based ensemble strategy was implemented to integrate the outputs of two classifiers (i.e., the ResNet and the CNN + MLP).

As anticipated, both models output a scalar probability (*p*_1_ from ResNet and *p*_2_ from CNN + MLP) representing the estimated likelihood of IUGR. Rather than combining these probabilities through heuristic averaging, we adopted a stacking approach in which a meta-classifier is trained to learn the optimal combination of the two predictive streams.

Specifically, a logistic regression model was used as meta-learner. The input to the meta-classifier is the two-dimensional vector *x* *=* $\left[p_{1},p_{2}\right]$ and the output is a calibrated probability of IUGR (Equation 1):

$$\begin{array}{c} p_{E}=\;\sigma (\beta_{0}+\beta_{1}p_{1}+\beta_{2}p_{2}) \end{array}$$ (1)

where σ(z) is the logistic sigmoid function, defined as (Equation 2):

$$\begin{array}{c} \sigma (z)=\;\frac{1}{1+e^{-z}} \end{array}$$ (2)

with the parameters $\beta_{0}$, $\beta_{1}$ and $\beta_{2}$ being learned during training and with a clear probabilistic interpretation. The intercept $\beta_{0}$ defines the baseline log-odds of IUGR when both base-model probabilities are zero. The $\beta_{1}$ and $\beta_{2}$ coefficients respectively quantify how strongly the ensemble model should trust the model generating $p_{1}$ and $p_{2}$.

As will be detailed in Section 2.6, the ensemble meta-classifier is trained on a validation subset derived from the training cohort to prevent information leakage. The training process proceeds such that both trained models are applied to the validation set to obtain pairs $\left(p_{1,i},p_{2,i}\right)$ for each subject *i*. These probability pairs are used as input features to the logistic regression model. The three β parameters are estimated by maximizing regularized log-likelihood (Equation 3):

$$L(\;\beta )=\;\underset{i}{\sum }[y_{i}\log(p_{E,i})+(1-y_{i})log(1-p_{E,i})]$$ (3)

where $y_{i}\in \{0,\;1\}\;$ denotes the true label.

Through this process the meta-classifier learns how to weight the relative reliability of each base model. This suggests whether one model should dominate in high-confidence regimes, whether agreement between models strongly increases risk, or whether discordant predictions require attenuation.

To further support the choice of the meta-learner, an ablation study was performed comparing logistic regression with alternative meta-classification strategies. Specifically, mean probability, support vector machine (SVM) and random forest (RF) classifiers were evaluated using the same two-dimensional input space defined by the output probabilities of the ResNet and CNN + MLP models. The results of this comparison are reported in Section 3.4.

### 2.6 Data preparation for training and testing

For the purposes of the present study, we focused on pregnancies labelled as healthy or IUGR within the NAPAMI cohort. IUGR cases were subdivided into two categories: presumed IUGR, based on antenatal clinical suspicion, and confirmed IUGR, defined according to postnatal outcome information available at birth.

The data subset employed in this work comes from a selected cohort of NAPAMI, encompassing the best signals in terms of quality (signal artefacts corrupting <2% of FHR) with gestational weeks between 30 and 40. It includes 7,864 healthy recordings and 1,762 presumed IUGR recordings, whereas the confirmed subset included 1,121 healthy recordings and 580 confirmed IUGR recordings. The majority of presumed IUGR cases lacked postnatal confirmation and may therefore include fetuses that were constitutionally small for gestational age (SGA) but ultimately healthy at birth. Consequently, this group may contain a degree of diagnostic uncertainty and potential label noise. To account for this aspect, the proposed methodology adopted a two-stage training strategy in which both the dataset composition and the learning process were structured to reflect the progressive refinement of diagnostic certainty encountered in clinical practice. The same training strategy was adopted for both the ResNet and the hybrid CNN + MLP models.

Within the presumed subset, a 90–10 partition was applied. Ninety percent of the presumed data, corresponding to 7,078 healthy recordings and 1,586 presumed IUGR recordings, was used to train the two base models, namely the ResNet and CNN + MLP models. The remaining 10%, corresponding to 786 healthy recordings and 176 presumed IUGR recordings, was reserved exclusively for training the ensemble meta-classifier.

Within the confirmed subset, a structured 80–10–10 subdivision was adopted. Eighty percent of the confirmed data, corresponding to 897 healthy recordings and 464 confirmed IUGR recordings, was used for fine-tuning the base models, allowing refinement of the internal representations using outcome-validated labels. Ten percent of the confirmed data, corresponding to 112 healthy recordings and 58 confirmed IUGR recordings, was reserved as an independent test set for final performance evaluation. The remaining 10%, corresponding to 112 healthy recordings and 58 confirmed IUGR recordings, was allocated to the training of the ensemble meta-classifier, enabling the meta-layer to learn from both presumed and confirmed examples while maintaining strict separation from the final test cohort.

The models were trained for up to 40 epochs using the Adam optimizer with an initial learning rate of 3 × 10^−^⁴ and weight decay of 10^−^⁴. Early stopping based on validation loss, with a patience of 8 epochs, was employed to prevent overfitting. During the fine-tuning stage on confirmed cases, the learning rate was reduced to 10^−^⁴ and training was performed for up to 15 epochs. To further ensure robustness and reduce variance associated with a single data split, a stratified 10-fold cross-validation procedure was performed within the development subsets, preserving the class distribution at the patient level in each fold. Model selection and hyperparameter tuning were applied exclusively within these cross-validation loops, while the independent confirmed test set remained entirely untouched until final evaluation. All partitions were performed at the patient level, ensuring that recordings from the same pregnancy were never distributed across different subsets. This strategy prevented longitudinal data leakage and preserved independence between development and evaluation phases.

## 3 Results

The classification performance of the two base models (CNN + MLP and ResNet) and of the proposed stacking ensemble is summarized in Table 1. Both the base models achieved comparable performance levels. The CNN + MLP architecture obtained a sensitivity of 0.763 and a specificity of 0.760, whereas the ResNet model achieved a slightly higher specificity (0.780) while maintaining a comparable sensitivity (0.761). These results indicate that the two architectures extract complementary information from the CTG recordings, despite relying on substantially different input representations. The CNN + MLP model integrates physiologically interpretable quantitative descriptors and image-based encodings of the FHR signal, while the ResNet architecture operates directly on multivariate temporal sequences. The similar performance achieved by the two models suggests that both modelling strategies capture meaningful aspects of fetal physiological dynamics. The stacking ensemble combining the predictions of the two base models produced a clear improvement in overall performance. In particular, the ensemble achieved a specificity of 0.839 and a sensitivity of 0.762, corresponding to a balanced accuracy of 0.799. The improvement is primarily driven by a substantial reduction in false positives, while maintaining a stable detection rate for IUGR cases. To assess whether the performance improvement obtained with the ensemble was statistically significant, a McNemar test was conducted comparing the predictions of the ensemble against the two base models. The test revealed a statistically significant improvement for the ensemble classifier (*χ*^2^ = 47.27, *p* < 10^−11^), indicating that the observed performance gain cannot be attributed to random variation. This result confirms that the integration of heterogeneous modelling strategies through the stacking framework produces a meaningful improvement in classification accuracy.

**Table 1:** Classification performance.

Model	TN	FP	FN	TP
CNN + MLP	0.760	0.240	0.237	0.763
ResNet	0.780	0.220	0.239	0.761
Ensemble	**0.839**	**0.161**	**0.238**	**0.762**

Bold values indicate the top performing scores.

### 3.1 Confusion matrix analysis

The confusion matrix of the final ensemble model is reported in Table 2. The model correctly identified 83.9% of healthy pregnancies and 76.2% of IUGR cases. The resulting error distribution is relatively balanced and in clinical terms, this is an important feature, since excessive false negatives could delay the identification of fetuses at risk, whereas excessive false positives could trigger unnecessary diagnostic procedures or monitoring protocols. The ensemble classifier therefore seems to achieve a reasonable trade-off between sensitivity and specificity.

**Table 2:** Confusion matrix for the ensemble classifier.

		Predicted	
		Healthy	IUGR
True	Healthy	0.839	0.161
	IUGR	0.239	0.762

### 3.2 ROC and precision-recall analysis

**Figure 4:**
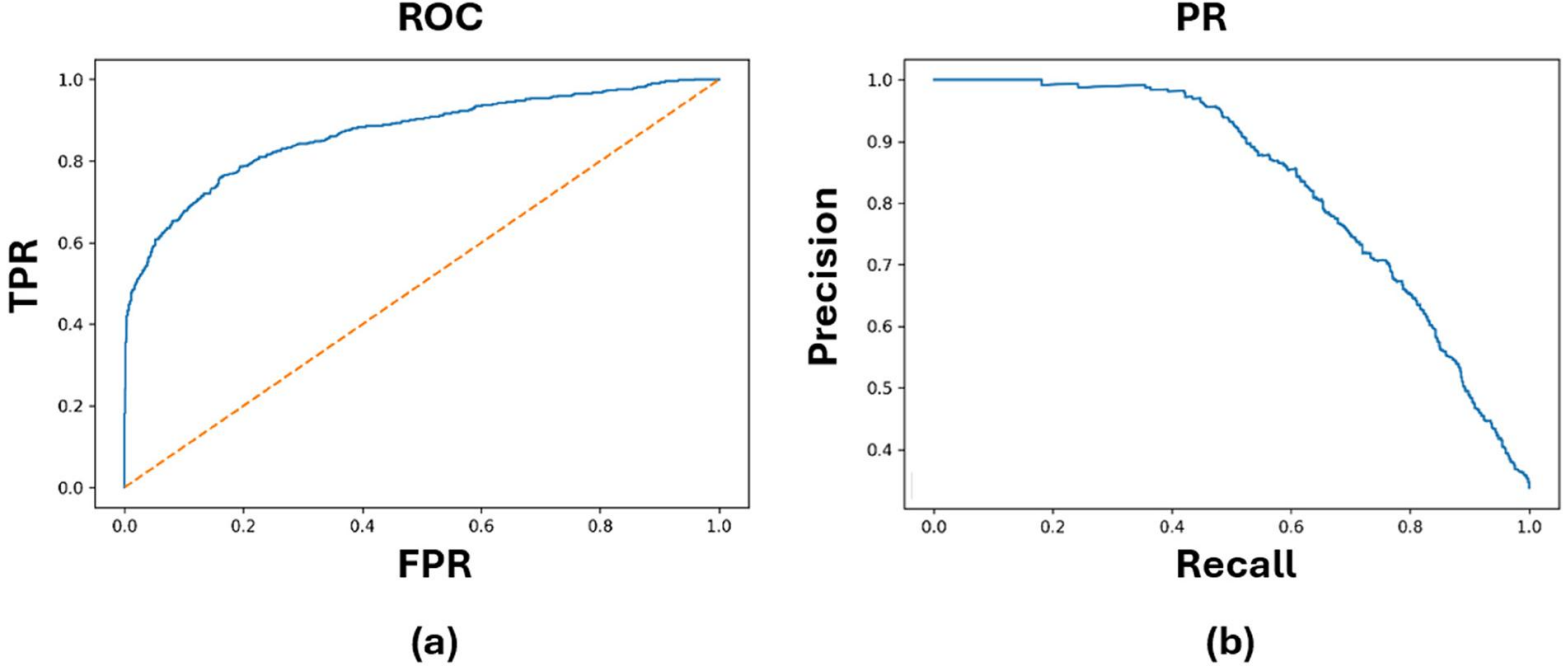
ROC curve **(a)**; precision-recall curve **(b)**.

The ROC curve of the ensemble classifier is shown in Figure 4a while the Precision–Recall (PR) curve is illustrated in Figure 4b. The ensemble model achieved an Area Under the ROC Curve (AUC) of 0.8676, indicating strong discrimination between IUGR and healthy pregnancies. Across the entire range of operating thresholds, the ensemble consistently maintains higher true positive rates for a given false positive rate compared with the individual base models. The smooth shape of the ROC curve and the narrow bootstrap confidence interval (95% CI: 0.8493–0.8854) indicate stable discrimination performance across resampled subsets of the test population. This suggests that the model generalizes well and is not overly sensitive to the specific composition of the test set.

The Precision–Recall (PR) curve further supports these findings. In particular, the ensemble maintains higher precision values at comparable recall levels, indicating that the predicted IUGR cases are more reliable and less affected by false alarms. This behavior is consistent with the improved specificity observed in the confusion matrix and highlights the benefit of integrating heterogeneous modelling approaches within a unified ensemble framework.

### 3.3 Bootstrap performance estimates

To quantify the robustness of the ensemble model, performance metrics were estimated using bootstrap resampling. The resulting 95% confidence intervals are reported in Table 3.

**Table 3:** Ensemble model performance scores.

Metric	Value with 95% CI
AUC	**0.868** (0.8493–0.8854)
BACC	**0.799** (0.7794–0.8186)
Sensitivity	**0.762** (0.7264–0.7913)
Specificity	**0.839** (0.8187–0.8583)
*F*1-score	**0.732** (0.7037–0.7577)
MCC	**0.588** (0.5483–0.6270)

Bold values indicate the average score.

The relatively narrow confidence intervals across all metrics indicate stable model behaviour and limited variance across resampled datasets. In particular, the MCC value of 0.588 reflects a moderate-to-strong overall correlation between predicted and true labels, confirming the reliability of the classifier even in the presence of class imbalance.

Overall, these results demonstrate that the proposed ensemble pipeline provides robust and consistent predictive performance across multiple evaluation metrics.

### 3.4 Ensemble meta-classifier interpretation

Table 4 reports the performance metrics of Logistic Regression against the other tested meta-classifiers. Logistic regression achieved the highest performance across all computed metrics (BACC = 0.799, AUC = 0.868, *F*1-score = 0.732, MCC = 0.588) with a significative level *p* < 0.05.

**Table 4:** Comparison of diverse meta-classifiers.

Meta-learner	BACC	AUC	*F*1-score	MCC
Mean probability	0.789	0.859	0.714	0.571
Random forest	0.793	0.863	0.726	0.575
RBF-SVM	0.796	0.865	0.729	0.580
Logistic regression	**0** **.** **799**	**0** **.** **868**	**0** **.** **732**	**0** **.** **588**

Bold values indicate the top performing scored.

The logistic regression meta-classifier's learnt coefficients are summarized in Table 5:

**Table 5:** Meta classifier's learnt coefficients.

Parameter	Value
*β*_0_	−1.2
*β*_1_ (ResNet)	1.8
*β*_2_ (CNN + MLP)	1.7

The positive values of *β*_1_ and *β*_2_ indicate that both base models contribute positively to the final decision. The similar magnitude of the two coefficients suggests that the meta-classifier assigns comparable importance to the predictions of the two architectures. This result further supports the hypothesis that the two modelling branches provide complementary but equally informative perspectives on the CTG signal.

### 3.5 Calibration analysis

**Figure 5:**
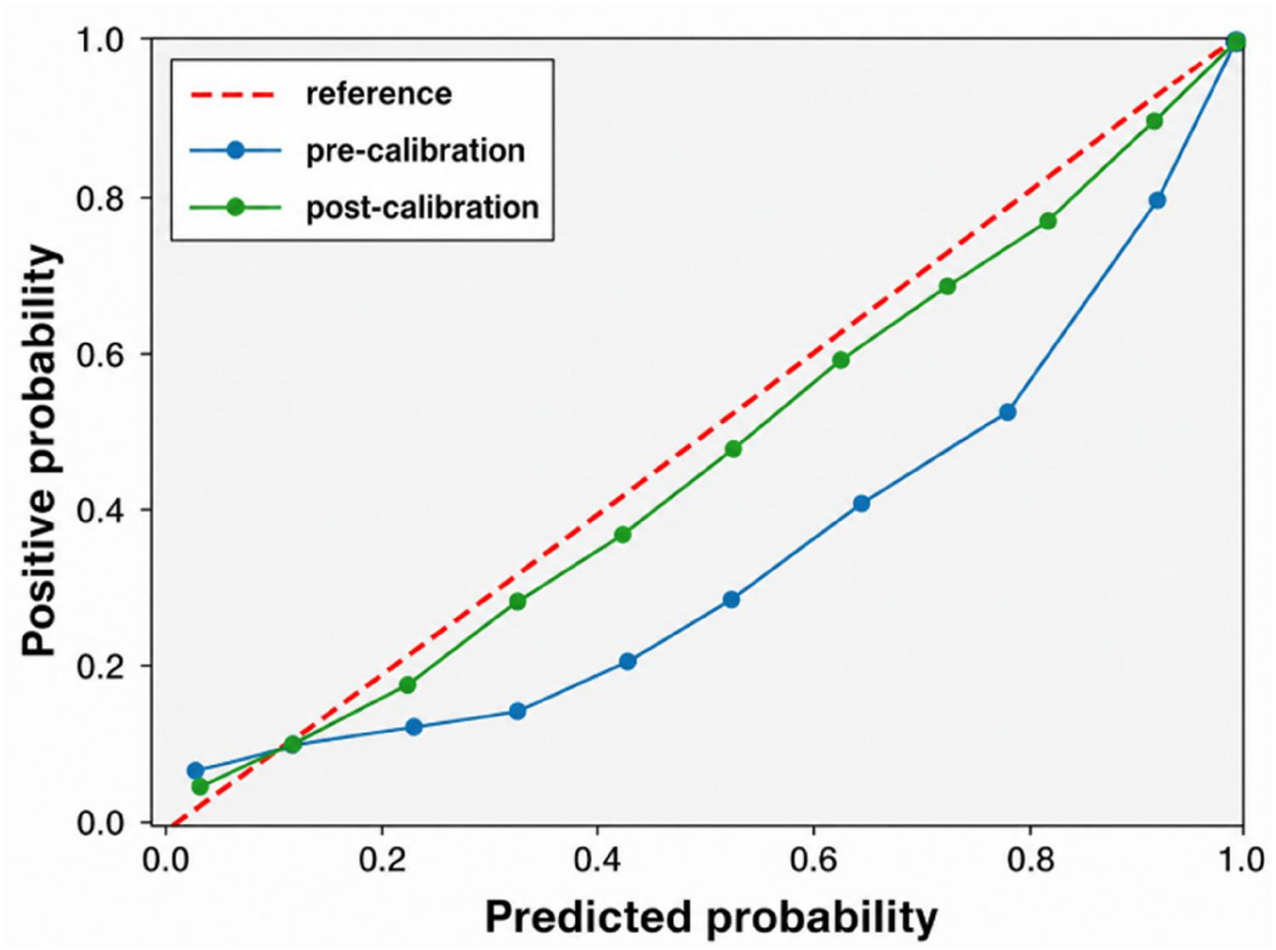
Calibration curve for the ensemble model.

Calibration analysis was performed to assess the agreement between predicted probabilities and observed outcome frequencies. The calibration curves before and after *post-hoc* calibration are shown in Figure 5, while Table 6 reports the corresponding quantitative calibration metrics.

**Table 6:** Quantitative calibration metrics.

Metric	Before calibration	After calibration
Brier score	0.158	0.145
ECE	0.086	0.041
CI	−0.31	−0.08
CS	0.72	0.91

Before *post-hoc* calibration, the ensemble model showed moderate overconfidence, particularly in the intermediate probability range. This was reflected by a Brier score of 0.158 and an expected calibration error (ECE) of 0.086. The Brier score ranges from 0 to 1, with lower values indicating better probabilistic accuracy, while the ECE quantifies the average discrepancy between predicted probabilities and observed event frequencies across probability bins, with 0 representing perfect calibration. After *post-hoc* calibration, probabilistic reliability improved, with the Brier score decreasing to 0.145 and the ECE decreasing to 0.041, indicating a lower overall prediction error and an approximately two-fold reduction in the average calibration gap.

Calibration intercept (CI) and calibration slope (CS) were also computed to further characterize model calibration. The ideal calibration intercept is 0, indicating no systematic overestimation or underestimation of risk, while the ideal calibration slope is 1. A slope lower than 1 is generally associated with overconfident predictions, whereas a slope higher than 1 suggests underconfident predictions. Before calibration, the model showed a calibration intercept of −0.31 and a calibration slope of 0.72, consistent with moderate overconfidence. After *post-hoc* calibration, both metrics moved closer to their ideal values, with the calibration intercept improving to −0.08 and the calibration slope increasing to 0.91. Overall, these results indicate a moderate but consistent improvement in probability calibration after *post-hoc* correction.

## 4 Discussion

The results of the present study show that the proposed multidimensional ensemble pipeline can effectively discriminate pregnancies complicated by IUGR from physiological pregnancies using antepartum CTG recordings collected in routine clinical practice.

The two base models achieved comparable standalone performance, with the CNN + MLP reaching a sensitivity of 0.763 and a specificity of 0.760, and the ResNet achieving a sensitivity of 0.761 and a slightly higher specificity of 0.780. When their outputs were combined through the stacking-based meta-classifier, the overall performance improved, reaching a balanced accuracy of 0.799, an AUC of 0.868 (95% CI: 0.849–0.885), a sensitivity of 0.762, a specificity of 0.839, an *F*1-score of 0.732 and an MCC of 0.588. The gain was mainly driven by a marked reduction in false positives, while preserving substantially unchanged sensitivity. This improvement was statistically supported by McNemar's test, which confirmed that the ensemble significantly outperformed the individual base models (*χ*^2^ = 47.27, *p* < 10^−11^).

These results are better than from a methodological perspective, these findings support the hypothesis that the two modelling branches extract complementary information from the CTG signal. The ResNet operates directly on multichannel temporal sequences and is therefore able to learn hierarchical temporal representations in a fully data-driven manner. In contrast, the hybrid CNN + MLP branch combines image-based encodings of FHR dynamics with structured quantitative descriptors derived from time-domain, spectral and nonlinear analysis, including state-dependent features computed within fetal activity and quiescence phases. The fact that the meta-classifier assigned comparable weights to the two base predictors (*β*_1_ = 1.8, *β*_2_ = 1.7) suggests that neither branch dominates the decision process and that both provide relevant and non-redundant information for classification.

An important aspect of the proposed framework is that the MLP branch of the hybrid model is built on physiologically related descriptors of fetal autonomic regulation. This does not make the model fully transparent, but it allows the classification process to be interpreted in a less purely black-box fashion, since part of the predictive decision is grounded in components with a known meaning. In this sense, the model not only learns discriminative patterns directly from the data, but also incorporates a structured representation of fetal physiology through a set of well-assessed indices, which may help relate classification performance to biologically meaningful signal properties.

The predictive performance obtained in this study is broadly consistent with the current state of the art, while being achieved in a more challenging, heterogeneous and clinically realistic setting. As anticipated in the Introduction, although several deep learning studies (both on generic healthy-vs.-pathological CTG classification and on IUGR prediction) reported higher performance than those obtained in the present work, these results are often derived from substantially smaller and more curated datasets (37, 46). In many reported cases, the analyzed cohorts are strongly imbalanced and neonatal outcome information is not available in the dataset. Consequently, labels rely exclusively on clinical interpretation of CTG tracings rather than on diagnoses confirmed at birth.

Nevertheless, our dataset is not completely free from this limitation. A considerable fraction of the recordings corresponds to pregnancies classified as presumed IUGR during antenatal monitoring, for which postnatal confirmation is not always available. However, unlike many previously published datasets, NAPAMI also includes a substantial number of cases linked to neonatal outcomes at birth.

To address this partial label uncertainty, we adopted a structured two-stage training strategy. In the first stage, the models were trained on the larger cohort of presumed cases in order to learn robust signal representations from a broad population. In the second stage, the models were fine-tuned using recordings associated with outcome-confirmed diagnoses, allowing the decision boundaries to be refined based on labels with higher diagnostic reliability.

This training paradigm also reflects the actual clinical workflow, in which suspicion is formulated during antepartum monitoring whereas confirmation becomes available only after birth. At the same time, it highlights one of the central limitations of current AI applications in prenatal medicine: the quality of the target labels. A proportion of presumed IUGR recordings may correspond to constitutionally small but healthy fetuses (Small for Gestational Age—SGA), thereby introducing label noise and reducing the effective separability of the two classes. Improving the certainty and granularity of outcome definitions may therefore represent a key factor for further improving model performance, for example through systematic linkage with neonatal outcome registries, birthweight centiles, Doppler-confirmed placental insufficiency, cord blood gas analysis, and postnatal clinical follow-up.

Calibration analysis provides further insight in this direction. The ensemble showed acceptable probabilistic behavior, with a Brier score of 0.145, but the calibration curve revealed moderate overconfidence in the intermediate probability range. This suggests that the model is already able to rank subjects reliably, as confirmed by the AUC and MCC values, although the estimated probabilities could still be made more clinically reliable. Improving outcome certainty and label quality may therefore contribute not only to higher discrimination performance but also to better calibration of the predicted risk.

## 5 Conclusion

This study proposes a multidimensional ensemble pipeline for the detection of IUGR from antepartum CTG recordings collected in routine clinical practice. The obtained results suggest that combining data-driven temporal representations with physiologically informed features (both related to a specific fetal behavioral state or neither) can enhance the robustness of AI-based CTG interpretation for IUGR risk assessment. The study also highlights the importance of explicitly addressing label uncertainty in prenatal AI applications and the adoption of a two-stage training strategy allowed the model to exploit a large cohort of presumed IUGR cases while refining its decision process on outcome-confirmed diagnoses.

Future developments should first address the validation of the proposed framework on additional clinically curated antepartum CTG cohorts with comparable IUGR-specific annotations. This aspect is particularly important because most publicly available CTG datasets are either intrapartum, based on generic fetal pathological outcomes, or represented by precomputed features rather than raw antepartum CTG signals, limiting their suitability for a direct external validation of the present task. Therefore, future validation should preferably rely on independent datasets specifically designed for antepartum IUGR assessment and including reliable postnatal outcome information.

Further analyses should also investigate the physiological contribution of the state-dependent features in greater detail. Although the present model already incorporates a validity-mask strategy to handle recordings lacking one behavioral phase, future studies on larger cohorts could assess whether the availability, duration, and distribution of Activity and Quiescence phases carry independent prognostic information. This would allow the relationship between fetal behavioral-state expression and IUGR risk to be explored more directly, beyond its current use as a structured feature-extraction framework.

Finally, additional work should focus on improving the clinical interpretability and reliability of the ensemble output. This may include more detailed analyses of the relative contribution of the ResNet and CNN + MLP branches, evaluation of alternative meta-classifiers when larger validation cohorts become available, and dedicated calibration strategies to reduce overconfidence in intermediate-risk predictions. Together, these developments would help clarify not only whether the model performs well, but also under which clinical and physiological conditions its predictions are most reliable.

## Data Availability

Data are available upon request to the authors. Requests to access these datasets should be directed to Giovanni Magenes (giovanni.magenes@unipv.it) and Maria Gabriella Signorini (mariagabriella.signorini@polimi.it).

## References

[1] American College of Obstetricians and Gynecologists. Antepartum fetal surveillance. Obstet Gynecol. (2021) 137:e116–27. 10.1097/AOG.0000000000004410 34011889

[2] Grivell RM Alfirevic Z Gyte GM Devane D. Antenatal cardiotocography for fetal assessment. Cochrane Database Syst Rev. (2015) 9:CD007863. 10.1002/14651858.CD007863.pub4 PMC6510058 26363287

[3] Hoyer D Żebrowski J Cysarz D Molnar BWJ Kusumoto R Heuser BJJW. Monitoring fetal maturation—objectives, techniques and indices of autonomic function. Physiol Meas. (2017) 38:R61–88. 10.1088/1361-6579/aa5fca 28186000 PMC5628752

[4] Alfirevic Z Gyte GML Cuthbert A Devane D. Continuous cardiotocography (CTG) as a form of electronic fetal monitoring (EFM) for fetal assessment during labour. Cochrane Database Syst Rev. (2017) (2):CD006066. 10.1002/14651858.CD006066.pub3. PMC6464257 28157275

[5] van Geijn HP. Developments in CTG analysis. Baillieres Clin Obstet Gynaecol. (1996) 10:185–209. 10.1016/S0950-3552(96)80033-2 8836480

[6] de Haan J van Bemmel JH Stolte LAM. Quantitative evaluation of fetal heart rate patterns. Eur J Obstet Gynecol Reprod Biol. (1971) 1:95–102. 10.1016/0028-2243(71)90056-6

[7] Arduini D Rizzo G Romanini C. Computerized analysis of fetal heart rate. J Perinat Med. (1994) 22(Suppl 1):22–7. 10.1515/jpme.1994.22.s1.22 7931996

[8] Dawes GS Moulden M Redman CWG. System 8000: computerized antenatal fetal heart rate analysis. J Perinat Med. (1991) 19:47–51. 10.1515/jpme.1991.19.1-2.47 1870056

[9] Pardey J Moulden M Redman CWG. A computer system for the numerical analysis of nonstress tests. Am J Obstet Gynecol. (2002) 186:1095–103. 10.1067/mob.2002.122444 12015543

[10] Task Force of the European Society of Cardiology and the North American Society of Pacing and Electrophysiology. Heart rate variability: standards of measurement, physiological interpretation, and clinical use. Circulation. (1996) 93:1043–65. 10.1161/01.CIR.93.5.1043 8598068

[11] Sassi R Cerutti S Lombardi F Malik M Huikuri HV Peng C-K. Advances in heart rate variability signal analysis. Europace. (2015) 17:1341–53. 10.1093/europace/euv015 26177817

[12] Signorini MG Magenes G Cerutti S Arduini D. Linear and nonlinear parameters for the analysis of fetal heart rate signal from cardiotocographic recordings. IEEE Trans Biomed Eng. (2003) 50(3):365–74. 10.1109/TBME.2003.808824 12669993

[13] Ferrario M Signorini MG Magenes G Cerutti S. Comparison of entropy-based regularity estimators. IEEE Trans Biomed Eng. (2006) 53:119–25. 10.1109/TBME.2005.859809 16402611

[14] Ferrario M Signorini MG Magenes G. Complexity analysis of the fetal heart rate variability. Med Biol Eng Comput. (2009) 47:911–19. 10.1007/s11517-009-0502-8 19526262 PMC2734261

[15] Martins JG Biggio JR Abuhamad A. Society for maternal-fetal medicine consult series #52: diagnosis and management of fetal growth restriction: (replaces clinical guideline number 3, April 2012). Am J Obstet Gynecol. (2020) 223(4):B2–17. 10.1016/j.ajog.2020.05.010 32407785

[16] Gordijn SJ Beune IM Thilaganathan B Papageorghiu A Baschat AA Baker PN. Consensus definition of fetal growth restriction. Ultrasound Obstet Gynecol. (2016) 48:333–9. 10.1002/uog.15884 26909664

[17] Esposito G Pini N Tagliaferri S Campanile M Zullo F Magenes G. Integrated approach based on advanced CTG parameters and Doppler measurements. BMC Pregnancy Childbirth. (2021) 21:775. 10.1186/s12884-021-04235-0 34784882 PMC8594236

[18] Esposito FG Tagliaferri S Giudicepietro A Giuliano N Maruotti GM Saccone G. Fetal heart rate monitoring and neonatal outcome. J Obstet Gynaecol Res. (2019) 45:1343–51. 10.1111/jog.13970 31099119

[19] Ferrario M Signorini MG Magenes G. Comparison between fetal heart rate standard parameters and complexity indexes. Methods Inf Med. (2007) 46:186–90. 10.1055/s-0038-1625404 17347753

[20] Tagliaferri S Fanelli A Esposito G Esposito FG Magenes G Signorini MG. Evaluation of phase-rectified slope. Comput Math Methods Med. (2015) 2015:236896. 10.1155/2015/236896 26779279 PMC4687338

[21] Signorini MG Pini N Malovini A Bellazzi R Magenes G. Integrating machine learning techniques and physiology-based features. Comput Methods Programs Biomed. (2020) 185:105015. 10.1016/j.cmpb.2019.105015 31678794

[22] Signorini MG Pini N Malovini A Bellazzi R Magenes G. Dataset on linear and non-linear indices. Data Brief. (2020) 29:105206. 10.1016/j.dib.2020.105206 32071962 PMC7015997

[23] Magboo VPC Magboo MSA. Prediction of late IUGR using machine learning. Procedia Comput Sci. (2022) 207:1427–36. 10.1016/j.procs.2022.09.200

[24] Petrozziello A Redman CWG Papageorghiou AT Jordanov I Georgieva A. Multimodal convolutional neural networks. IEEE Access. (2019) 7:112026–36. 10.1109/ACCESS.2019.2933448

[25] Ogasawara J Ikenoue S Yamamoto H Sato M Kasuga Y Mitsukura Y. Deep neural network-based classification of cardiotocograms. Sci Rep. (2021) 11:13367. 10.1038/s41598-021-92805-9 34183748 PMC8238938

[26] Cao Z Wang G Xu L Li C Hao Y Chen Q. Intelligent antepartum fetal monitoring via deep learning. Health Inf Sci Syst. (2023) 11:16. 10.1007/s13755-023-00207-5 36950107 PMC10025176

[27] Zhao Z Deng Y Zhang Y Zhang Y Zhang X Shao L. DeepFHR: prediction of fetal acidemia. BMC Med Inform Decis Mak. (2019) 19:286. 10.1186/s12911-019-0975-1 31888592 PMC6937790

[28] Fei Y He L Chen J Hao Y Liu G Chen Q. Classification via multimodal bidirectional GRU. Biomed Signal Process Control. (2022) 78:104008. 10.1016/j.bspc.2022.104008

[29] Mendis L Palaniswami M Keenan E Brownfoot F. Rapid detection of fetal compromise. Sci Rep. (2024) 14:63108. 10.1038/s41598-024-63108-6 PMC11144251 38824217

[30] Zhao Z Zhang Y Comert Z Deng Y. Diagnosis system incorporating recurrence plot with CNN. Front Physiol. (2019) 10:255. 10.3389/fphys.2019.00255 30914973 PMC6422985

[31] Zhou Z Zhao Z Zhang X Zhang X Jiao P Ye X. Identifying fetal status with deep learning. Comput Biol Med. (2023) 159:106970. 10.1016/j.compbiomed.2023.106970 37105114

[32] Kong L Snášel V Bai Z Vilimek D Mirjalili S Pan JS. Enhancing CTG classification via ensemble learning. Sci Rep. (2025) 15:38528. 10.1038/s41598-025-18990-z 41188245 PMC12586662

[33] Chudáček V Spilka J Burša M Janků P Hruban L Huptych M. Open access intrapartum CTG database. BMC Pregnancy Childbirth. (2014) 14:16. 10.1186/1471-2393-14-16 24418387 PMC3898997

[34] Ahmed S Mahmoud M. Early detection of fetal health status using AI. Neural Comput Appl. (2025) 37:16753–79. 10.1007/s00521-025-11343-x

[35] Khan MJ Vatish M Davis Jones G. PatchCTG: transformer for fetal monitoring. Sensors. (2025) 25:2650. 10.3390/s25092650 40363088 PMC12074329

[36] Mohan PPA Uma V Sasirekha R Hamsika V. FHR signal analysis using attention-based 1DCNN-BiLSTM neural network for intrapartum fetal monitoring. Digit Signal Process. (2025) 164:105259. 10.1016/j.dsp.2025.105259

[37] Rescinito R Ratti M Payedimarri AB Panella M. Prediction models for IUGR: systematic review. Healthcare. (2023) 11:1617. 10.3390/healthcare11111617 37297757 PMC10252230

[38] Xie W Cai P Hu Y Lu Y Chen C Cai Z. AI-driven paradigm shift in CTG analysis. Neurocomputing. (2024) 607:128446. 10.1016/j.neucom.2024.128446

[39] UCI Machine Learning Repository. Cardiotocography dataset (2010). Available online at: https://archive.ics.uci.edu/dataset/193 (Accessed December 22, 2025).

[40] Ayres-de Campos D Bernardes J Garrido A Marques-de Sa J Pereira-Leite L. Sisporto 2.0. J Matern Fetal Med. (2000) 9:311–18. 10.1002/1520-6661(200009/10)9:5<311::AID-MFM12>3.0.CO;2-9 11132590 10.1002/1520-6661(200009/10)9:5<311::AID-MFM12>3.0.CO;2-9

[41] Mendis L Karmakar D Palaniswami M Brownfoot F Keenan E. Cross-database evaluation of DL methods. IEEE J Transl Eng Health Med. (2025) 13:123–35. 10.1109/JTEHM.2025.3548401 40657532 PMC12250915

[42] Spairani E Daniele B Magenes G Signorini MG. A novel large structured cardiotocographic database. Annu Int Conf IEEE Eng Med Biol Soc. (2022) 2022:1375–8. 10.1109/EMBC48229.2022.9871340 36086045

[43] Spairani E Steyde G Spuri Forotti F Magenes G Signorini MG. Prediction of IUGR using ResNet. Comput Biol Med. (2025) 190:110123. 10.1016/j.compbiomed.2025.110123 40184939

[44] Spairani E Daniele B Signorini MG Magenes G. Deep learning mixed-data approach for the classification of FHR signals. Front Bioeng Biotechnol. (2022) 10:887549. 10.3389/fbioe.2022.887549 36003538 PMC9393210

[45] Spairani E Steyde G Tagliaferri S Signorini MG Magenes G. Fetal states identification via HMM. Comput Methods Programs Biomed. (2023) 240:107736. 10.1016/j.cmpb.2023.107736 37531691

[46] Steyde G Subitoni L Spairani E Magenes G Signorini MG. Siamese neural networks for IUGR identification in cardiotocographic recordings. Comput Cardiol. (2024) 51:1–4. 10.22489/CinC.2024.280

